# Magnetically Bioprinted Human Myometrial 3D Cell Rings as A Model for Uterine Contractility

**DOI:** 10.3390/ijms18040683

**Published:** 2017-03-23

**Authors:** Glauco R. Souza, Hubert Tseng, Jacob A. Gage, Arunmani Mani, Pujan Desai, Fransisca Leonard, Angela Liao, Monica Longo, Jerrie S. Refuerzo, Biana Godin

**Affiliations:** 1 Nano3D Biosciences, Houston, TX 77030, USA; GSouza@n3dbio.com (G.R.S.); HTseng5@gmail.com (H.T.); JGage@n3dbio.com (J.A.G.); PujanDesai@gmail.com (P.D.); Angela_Liao@sbcglobal.net (A.L.); 2 Division of Maternal-Fetal Medicine, Department of Obstetrics, Gynecology, and Reproductive Medicine, University of Texas Health Science Center at Houston, Houston, TX 77030, USA; Arunmani.Mani@uth.tmc.edu (A.M.); Monica.Longo@uth.tmc.edu (M.L.); Jerrie.S.Refuerzo@uth.tmc.edu (J.S.R.); 3 Department of Nanomedicine, Houston Methodist Research Institute, Houston, TX 77030, USA; FLeonard@houstonmethodist.org

**Keywords:** uterine contractility, tissue bio-printing, contractility assay, myometrium, patient-derived, tocolytics, personalization of therapy

## Abstract

Deregulation in uterine contractility can cause common pathological disorders of the female reproductive system, including preterm labor, infertility, inappropriate implantation, and irregular menstrual cycle. A better understanding of human myometrium contractility is essential to designing and testing interventions for these important clinical problems. Robust studies on the physiology of human uterine contractions require in vitro models, utilizing a human source. Importantly, uterine contractility is a three-dimensionally (3D)-coordinated phenomenon and should be studied in a 3D environment. Here, we propose and assess for the first time a 3D in vitro model for the evaluation of human uterine contractility. Magnetic 3D bioprinting is applied to pattern human myometrium cells into rings, which are then monitored for contractility over time and as a function of various clinically relevant agents. Commercially available and patient-derived myometrium cells were magnetically bioprinted into rings in 384-well formats for throughput uterine contractility analysis. The bioprinted uterine rings from various cell origins and patients show different patterns of contractility and respond differently to clinically relevant uterine contractility inhibitors, indomethacin and nifedipine. We believe that the novel system will serve as a useful tool to evaluate the physiology of human parturition while enabling high-throughput testing of multiple agents and conditions.

## 1. Introduction

The uterus is an organ of the female reproductive system. It is a hollow organ and has three main layers: a well-differentiated endometrium lining, a thick smooth muscle, known as “myometrium”, and an outer serosal layer [1,2,3]. The myometrium is the main layer responsible for uterine contractions. Uterine contractions are very important for multiple reproductive functions, such as the menstrual cycle, the transport of sperms and embryo, pregnancy, and parturition [4,5]. Deregulation in uterine contractility can serve as a basis in common pathological disorders including preterm labor and premature birth, infertility, abnormal implantation, and irregular menstrual cycle [6,7,8]. Moreover, a coordinated activity of uterine myometrial cells is required for initiation and flow of a successful labor course [9,10]. On the other hand, if uterine contractility is impaired, it significantly affects the progression of normal labor [2]. 

In the past few decades, there has been progress in shedding more light on the physiology of endometrial functions in normal and pathological conditions. A better understanding of endometrial functions and their regulations resulted in the development of several important interventions in the areas of conception, contraception, and normalization of menstrual function [3]. However, although the importance of abnormal uterine contractility is well acknowledged, there has been rather insufficient research focusing on the role of the uterine myometrium in common disorders of the female reproductive system. A better understanding of human myometrium physiology and contractility is essential to designing and testing interventions that can prevent or treat the important clinical problems noted above. 

However, the challenge is to identify an assay that accurately and efficiently models uterine contractility. In vivo models are, in general, costly, low-throughput, and time-consuming [11], but more importantly, there are sharp differences between species in birthing patterns, reflecting different biological bases that render these assays poorly predictive [12]. Ex vivo tests, such as organ chamber systems, can be accurate predictors of uterine contractility, yet suffer from sample inconsistencies, scarcity, and equipment costs [13,14,15]. An in vitro cell culture model is a viable alternative that overcomes issues with not only scarcity and cost, but reproducibility and throughput. However, the majority of in vitro cell culture models are two-dimensional (2D) monolayers that poorly mimic native tissue environments. For one, the typical plastic or glass substrates are much stiffer than in vivo tissue, let alone uteri. These models also misrepresent extracellular matrix (ECM) structure and composition, as well as the cell–cell and cell–ECM interactions that the ECM supports. Lastly, biochemical and nutrient access is far different in 2D than in vivo, as every cell is uniformly exposed to the surrounding environment [16,17,18]. 

A potential solution lies in the three-dimensional (3D) cell culture models that can recreate tissue structure and ECM composition in vitro. A variety of different 3D cell culture platforms exist: protein gels, such as Matrigel^®^ (BD Biosciences, San Jose, CA, USA) and collagen that recreate ECM composition [19]; polymer scaffolds that reproduce tissue structure and material properties [20]; hanging drop spheroids that use water tension in liquid droplets to aggregate cells into spheroids [21,22]; round bottom plates that use plate geometry to aggregate cells in spherical bottom wells [23]; and nano-patterned plates, with wells <1 µm patterned within the well where cells aggregate. While these systems approximate tissue environments to varying degrees, they have technical and cost limitations, such as long fabrication times, specialized equipment, and either attachment to stiff substrates that influence cell behavior or detachment that makes 3D cultures difficult to handle. Moreover, these platforms can only create spheroids—not more complex patterns—such as rings or strips that would better approximate uterine smooth muscle contractility. Collagen gels could be a solution to recapitulate the collagenous myometrium in cylindrical or strip form, but these gels require a complex fabrication process that limits throughput. Thus, there is a need for a 3D cell culture platform that can create complex patterns with speed and ease to recapitulate uterine contractility in vitro.

To fill in these gaps in our knowledge of uterine physiology, we are proposing here, for the first time, a three-dimensional (3D) in vitro model of human uterine myometrial cells for the evaluation of baseline uterine contractility physiology and as a function of various pathological conditions affecting pregnant woman’s health. The human myometrial cells are magnetically bio-printed in hollow rings (similarly to the cross-sections of the uterus, a hollow organ) and their contractility is evaluated over time and as a function of tocolytic agents that have been shown to affect the uterine contractions clinically. We believe that the novel system introduced in this work will serve as a valuable tool for the evaluation of physiology of human uterine contractions with the ability for high-throughput testing of multiple agents and conditions simultaneously. 

## 2. Results

### 2.1. Bioprinting Commercially Available Human Myometrium Cells

**Figure 1:**
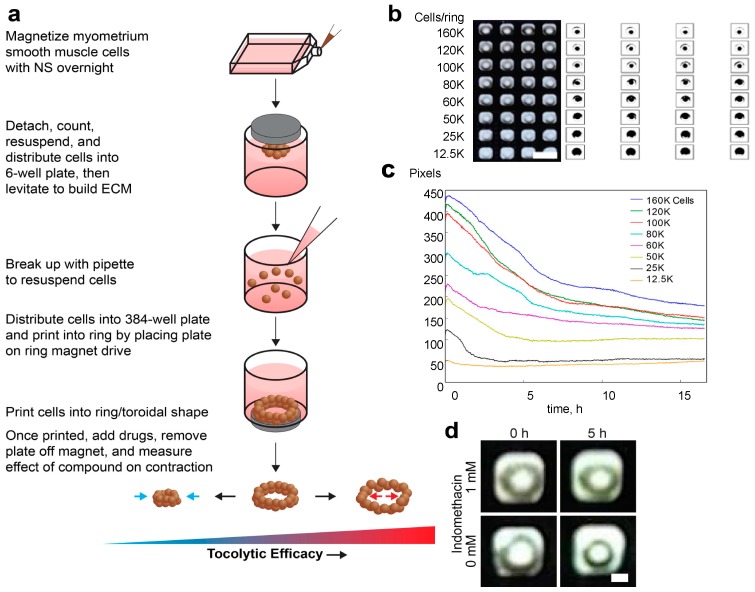
(**a**) Schematic presentation of the process flow of the proposed 3D uterine contractility assay; NS: nanoshuttle; ECM: extracellular matrix (**b**) Myometrial smooth muscle cell (SMC)-A printed at different cell densities as captured by the iPod (Apple Computer, Cupertino, CA, USA) (left), and their area measured by the Python-based analytical software (right). Full rings were detectable by the software starting at 100,000 cells (100 K)/ring or 40,427 cells/mm^2^, which was used as the cell density for this assay. Scale bar = 5 mm. (**c**) The contraction of SMC-A ring area as measured in pixels as a function of time at various cell densities. The rings contracted immediately after printing, suggesting that the levitation and printing times of 2 and 1 h, respectively, were sufficient to produce a contractile ring. Cell/ring values in legend are in thousands of pixels. (**d**) Tocolytic effect of indomethacin on myometrial SMC contractility, as detected with iPod-driven imaging system, scale bar = 1 mm.

In order to design an in vitro system for the evaluation of human uterine (myometrial) contractility, we initially determined the optimal conditions of the assay, including the cell/ring (well) concentration, and the times of levitation and printing. Using commercially available smooth muscle cells (SMC)-A and SMC-B, we found that magnetized myometrial cells can be bioprinted into competent rings at or above 50,000 cells/ring. However, our imaging tools, namely iPod (Apple Computer, Cupertino, CA, USA) and analytical software, could only detect full rings with at least 100,000 cells/ring, due to the cell’s density. Thus, 100,000 cells/ring was the concentration used for the remainder of the experiments. Figure 1a schematically shows the process flow diagram and Figure 1b presents iPod images of SMC-A cells bioprinted with various cell densities. 

When we allowed these rings to contract, we found that they contracted immediately after printing (Figure 1c,d and Figure S1). This is in agreement with the nature of the smooth muscle cells and suggests that cells within the ring rearranged into a contractile state during levitation and printing, such that the ring could contract once the magnet was removed. These results show that the 2 h of levitation time and the 1 h of printing time were sufficient to print contractile rings as was also shown previously for endothelial smooth muscle cells and other cell types [24,25,26]. These parameters were used for subsequent experiments. 

### 2.2. Bioprinting Freshly Excised and Cryopreserved Patient-Derived Myometrium Cells

Next, we have tested the response of the bioprinted uterine rings to commonly used agents that inhibit uterine contractility. For this purpose, we used tocolytic drugs clinically to inhibit uterine contractions during preterm labor, thus preventing preterm birth [27]. The most commonly used tocolytics are indomethacin (acts by inhibiting prostaglandin production) and nifedipine (a calcium channel blocker). Ibuprofen was used as a control drug for two reasons: (1) it has no effect on uterine contractility in the clinical setting; and (2) it has been previously shown to have an effect on other smooth muscle cells [24]. The drugs were added to the 3D bioprinted myometrial rings in various concentrations and the cell ring contractility was detected. 

Myometrial smooth muscle cells derived from three patients were bioprinted into rings at a concentration of 1 × 10^5^ cells/well in a 384-well plate by first levitating the cells for 2 h and then by printing the cells into rings for 1 h as described above. 

**Figure 2:**
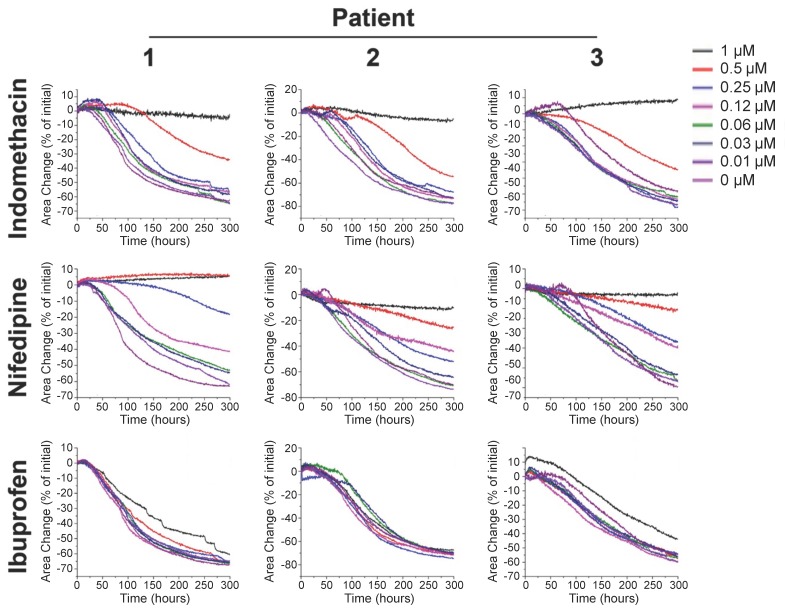
Contraction of myometrial smooth muscle rings over time. The contraction profiles of myometrial smooth muscle rings from different patients exposed to varying concentrations of different compounds. The myometrial smooth muscle rings were contractile as shown by the sudden drop in area of negative controls. As expected, clinically used tocolytics indomethacin, and nifedipine, had a inhibitory effect on myometrial smooth muscle rings contractility by slowing contraction, or in the case of both indomethacin and nifedipine, nearly stopping it. Overall, this assay detected dose-dependent effects on uterine contractility. Each concentration (dose) in the range of 0–1 μM is represented by a different color on the graphs as mentioned in the legend.

These rings were dosed with varying concentrations of the clinically used tocolytics, indomethacin and nifedipine, and ibuprofen, as a control. Once printed and removed off the magnet, these rings began to immediately contract, as evidenced by the quick drop in ring area in negative control rings (Figure 2). 

As can be seen from Figure 2, all tocolytics inhibited the contractions of the bioprinted myometrial rings and the time-response was concentration-dependent. Interestingly, different cells reacted differently to the three drugs. 

**Figure 3:**
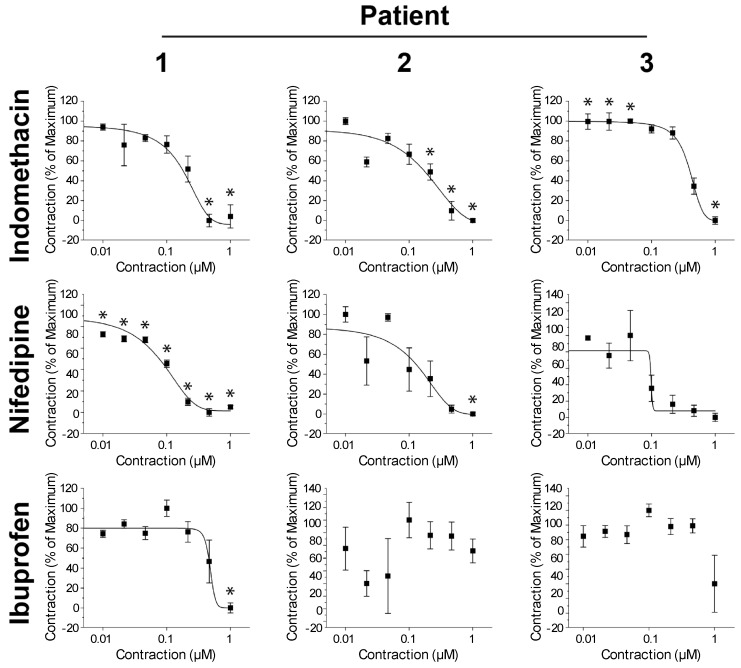
Dose responses of myometrial smooth muscle rings. The dose responses of myometrial smooth muscle cell rings to different tocolytics from different patients. The ring area changes after 2 h of contraction was used as the endpoint. The differences in dose response between patients serve to demonstrate the ability of this assay to detect patient-specific responses and tailor tocolytic therapy. * *p* < 0.05 vs. control. Error bars represent standard error.

Using the change in rings area after 2 h of contraction as the endpoint, a significant effect of concentration was found for the tocolytic agents but not for ibuprofen for Patients 2 and 3. As the data in Figure 3 show, indomethacin and nifedipine had relaxant effects on contraction, nearly stopping it. As expected, the dose responses and half-maximal inhibitory concentration (IC_50_) values varied between patients (Table 1). Overall, this new myometrial contractility assay is able to detected dose-dependent and patient-dependent uterine responses. 

We further tested cryopreserved cells from the same patients and found that, after one month, the responses to the tocolytic agents were maintained. As an example, Figure S2 shows a comparison in contractility time and dose-dependent profiles of patient-derived uterine cells.

## 3. Discussion

As mentioned above, robust studies to better understand the physiology of human uterine contractions cannot be performed in vivo and require in vitro models, which utilize human source. Moreover, since the contractility of the tissue is a phenomenon, which occurs in a three-dimensional environment enabling cell–cell interactions, it has to be evaluated in the same manner. In this work, we proposed and evaluated a 3D cell culture platform that could potentially overcome the above limitations to build a uterine contractility assay, which is based on magnetic 3D bioprinting [28,29,30,31]. The principle behind magnetic 3D bioprinting is the magnetization of cells, and their aggregation with magnetic forces to form and pattern 3D cell culture models. Cells are magnetized by incubation with a biocompatible nanoparticle assembly consisting of gold, iron oxide, and poly-l-lysine [31]. These cells can then be aggregated using magnetic forces, particularly into 3D patterns, such as spheroids or hollow rings, at the bottom of a well in a multi-well plate [24,25,28,29,30,31,32]. Once aggregated, these cells interact and build ECM to recapitulate native tissue environments [29,31]. Neither the nanoparticles nor the magnetic forces have any deleterious effects on cell behavior [24,25,26,27,28,29,30,31,32,33]. Using this platform, we have designed a uterine contractility assay that is simple yet robust and predictive. We successfully bioprinted myometrial rings in 384-well formats (Figure 1) and were able to follow up on changes in the ring area volume, which are the pharmacodynamics sign of the 3D structures contraction over time. Based on the smooth muscle cells physiology, bioprinted hollow myometrial rings were found to contract immediately after printing (Figure 1 and Figure S1). Exposure to tocolytic compounds, indomethacin and nifedipine, clinically used for inhibition of the myometrial contractions, affected the contraction in myometrial rings in a dose-dependent manner (Figure 2). On the other hand, the contractility of bioprinted rings to ibuprofen, which has no effect on uterine contractions in the clinic, was negligible. By testing varying dosages, an efficacy profile for the tocolytic drugs can be assessed (Figure 3 and Table 1). Using the same principle, we have previously developed assays for wound healing in rings [24] and toxicity in spheroids [32].

We have shown that the uterine rings can be bioprinted from primary cells obtained from patients during a cesarean section. It is noteworthy that, using a small tissue biopsy that can be easily obtained in a clinical setting, we were able to assess a multitude of agents and conditions in a high-throughput manner. These findings show the feasibility of using the 3D uterine contractility assay in the future for the personalization of therapies for uterine contractility disorders, such as preterm labor, infertility, inappropriate implantation, irregular menstrual cycles, and others. Moreover, while there is no doubt that this novel assay should be validated and compared to relevant clinical data, the fact that myometrium samples from various patients had different responses to various agents commonly used in clinical settings can shed more light on the individual physiology of the uterine contractions and the consequent pathologies related to the same. Interestingly, freezing had no significant effect on the contractility profiles, and freshly excised cells had similar dose and time response curves to those of the cells from the same patient that underwent a freezing and thawing cycle (Figure S2). 

In conclusion, we believe that, by using 3D bioprinting of human myometrial cells, we will address an unmet need for high-throughput evaluation of uterine contractility for basic, translational, and clinical research. We anticipate that this in vitro myometrial contractility assay can shed more light on the physiology of human uterine contractions and can be used as a valuable tool in the clinical setting to personalize therapies for uterine contractility disorders. 

## 4. Materials and Methods 

### 4.1. Cell Culture

#### 4.1.1. Commercially Available Human Uterine Smooth Muscle Cells

Human uterine smooth muscle cells (HUtSMCs) were obtained from PromoCell GmbH (Heidelberg, Germany). The cells are mentioned in the manuscript as SMC-A and SMC-B (PromoCell catalogue number C-12575 and C-12576, respectively). The cells were grown in PromoCell smooth muscle cell medium and 1% penicillin/streptomycin (P/S, Sigma, St. Louis, MO, USA). These cells were cultured in an incubator (37 °C, 5% CO_2_) with daily media exchange for the first 3 days, and every other day thereafter. Prior to magnetic bioprinting, the cells were assessed for viability (CellTiter-Glo, Promega, Madison, WI, USA).

#### 4.1.2. Patients-Derived Human Uterine Myometrial Cells

Primary human uterine smooth muscle cells (SMCs) were obtained from uterine biopsies from women undergoing scheduled cesarean section at term gestation (greater than 37 weeks of pregnancy) who have given written informed consent according to the Institutional Review Board (IRB)-approved protocol (University of Texas Health Science Center at Houston, HSC-MS-14-0370). Women with more than 3 contractions per hour, rupture of membranes, placenta previa, known infections, or uterine leiomyomas, and women under the age of 18, were excluded. Biopsies were taken from the upper edge of the lower segment of the transverse uterine incision (2 × 2 × 4 cm) and placed in Hank’s balanced salt solution (HBSS) without Ca^2+^ and Mg^2+^.

Preparation of the biopsies into a cell culture was then performed [34]. The biopsies were finely minced and digested into cells with 0.1% trypsin (Sigma) and 0.1% DNAse (Sigma) in HBSS for 30 min in a shaking incubator (37 °C). After centrifugation (400× *g* for 5 min), the enzymes were replaced with 0.2% collagenase Type I (Sigma) in HBSS to digest for another 30 min in a shaking incubator. The resulting cell and tissue suspension was then filtered and centrifuged, and the cells were resuspended in Roswell Park Memorial Institute (RPMI) 1640 medium (Sigma) with 10% fetal bovine serum (FBS, Sigma) and 1% penicillin/streptomycin (P/S). These cells were then seeded and cultured as explained previously for the primary human uterine smooth muscle cells [34].

### 4.2. Cryopreservation of Cells from Uterine Samples from Patients

For freezing (cryopreserving) the cells, we used different conditions: (a) flash freezing, in which the tissue was transferred immediately to a liquid nitrogen tank for long-term storage; (b) slow freezing, where the tissue was frozen stepwise at 4 °C for 20 min, −80 °C overnight, and then in liquid nitrogen; and (c) the cryobox method, in which the tissue was placed immediately into a CoolCell (Biocision, San Rafael, CA, USA) to freeze overnight at −80 °C, then transferred into liquid nitrogen. In all three cases, the cryoprotectant was 10% dimethylsulfoxide (DMSO) in SMC medium. The remaining unfrozen tissues were immediately harvested for cells as control. After one month of storage, the tissues were thawed, cryopreservation medium was replaced with HBSS without calcium and magnesium, the tissues were finely minced and prepared into a cell culture as described above. Based on the viability assay (CellTiter-Glo, Promega, Madison, WI, USA), we found that cryopreserving tissue using the cryobox method was the most efficient method to obtain >90% viable cells as compared to freshly processed tissues. Thus, further experiments with dose response agents related to cryopreserved (frozen) samples are based on this method. 

### 4.3. Magnetic 3D Bioprinting of Human Myometrial Cells

SMCs were magnetically 3D bioprinted into rings for this uterine contractility assay. SMCs were printed in a similar manner to a previous study using primary human tracheal SMCs [24]. Briefly, monolayers of SMCs at 70%–80% confluence were magnetized by adding a magnetic nanoparticle assembly (NanoShuttle, NS, Nano3D Biosciences, Houston, TX, USA) at a concentration of 1 µL/1 × 10^4^ cells for static incubation overnight. The method of the cell magnetization was previously described in details for other cell types [24,25,26]. The next day, the magnetized SMCs were detached, counted, and resuspended into cell-repellent 6-well plates (Greiner Bio-One, Frickenhausen, Germany) at a concentration of 3.2 × 10^6^ cells/well in 2 mL of media (1.6 × 10^6^ cells/mL). These SMCs were then levitated off the well bottom to aggregate and form an ECM endogenously by placing a magnetic levitation drive of six neodymium magnets atop the plate. Based on our prior publications with levitation and bioprinting of other cell types, ECM is being produced by the cells starting from 30 min of levitation [25,35]. After 2 h of levitation, the SMCs were resuspended in media and then redistributed into cell-repellent 384-well plates (Greiner Bio-One) at a concentration of 1 × 10^5^ cells/well in 80 µL of media (1.25 × 10^6^ cells/mL). We further used the levitation time of 2 h since, at a later time, SMCs formed very tight structures, which could not be dispersed for bioprinting. The SMC rings were then printed by placing the plate of cells atop a magnetic drive of 384 ring-shaped magnets (0.125′′ OD × 0.0625′′ ID) for 1 h to attract SMCs to the bottom of the well and form 1 ring/well. 

### 4.4. Myometrial Smooth Muscle Cell Ring Contractility Assay

The contraction of magnetically 3D bioprinted SMC rings was used to assess the efficacy of various tocolytics. After printing SMC rings, compounds, diluted from stock in media, were added to the wells. Three tocolytic drugs were tested at eight concentrations in triplicate: indomethacin (Sigma), nifedipine (Sigma), which are clinically used; and ibuprofen (Sigma), which served as control. Negative control wells had media with 1% DMSO (Sigma) added. Once the compounds were added, the plate of SMC rings was removed off the magnets to allow the cells to contract, and moved immediately to an imaging system utilizing a mobile device (iPod, Apple Computer, Cupertino, CA, USA), as was done in previous studies. The mobile device was programmed using an app (Experimental Assistant, Nano3D Biosciences) to image the plate every 30 s for 10 h. Once the SMC rings finished contracting, the images were moved from the mobile device to a separate computer, where they were batch-analyzed to measure ring area over time using custom image analysis software written in Python programming language. The endpoint used to assess the tocolytic dose responses was the contracting area change after 2 h and normalized between the maximum and minimum contractions. The dose response was fit to a sigmoidal curve (OriginPro, OriginLab, Northampton, MA, USA), and the IC_50_ was obtained from the curve. 

### 4.5. Statistical Analysis

The contraction data from the uterine smooth muscle cell ring assay was statistically analyzed using ANOVA tests (OriginPro, Northampton, MA, USA), both one-way for the effect of drug concentration, and two-way for the effects of drug concentration and cell source. Significance was defined as *p* < 0.05. Error bars represent standard error. 

## Figures and Tables

**Table 1:** Half-maximal inhibitory concentration (IC_50_) values in μM obtained from uterine smooth muscle ring assay using myometrial smooth muscle cells from different patients.

Drug	Patient 1	Patient 2	Patient 3
Indomethacin	0.18	0.17	0.40
Nifedipine	0.08	0.13	0.10
Ibuprofen	0.46	-	-

## Supplementary Materials

Supplementary materials can be found at www.mdpi.com/1422-0067/18/4/683/s1.

## Abbreviations

2D	two-dimensional
3D	three-dimensional
ECM	extracellular matrix
SMC	smooth muscle cells
NS	nano-shuttle
IC_50_	inhibitory concentration 50, concentration at which the contractility is 50% of the maximum
