# Supplementary material for: Magnetically Bioprinted Human Myometrial 3D Cell Rings as A Model for Uterine Contractility
Source: Int J Mol Sci. 2017 Mar 23;18(4):683. doi: 10.3390/ijms18040683 (PMC5412269; doi:10.3390/ijms18040683)
Supplement: Supplementary file 1 [file ijms-18-00683-s001.pdf]

Supplementary Materials: Magnetically Bioprinted Human Myometrial 3D Cell Rings as a Model for Uterine Contractility

Glauco R. Souza, Hubert Tseng, Jacob A. Gage, Arunmani Mani, Pujan Desai, Fransisca Leonard, Angela Liao, Monica Longo, Jerrie S. Refuerzo, Biana Godin

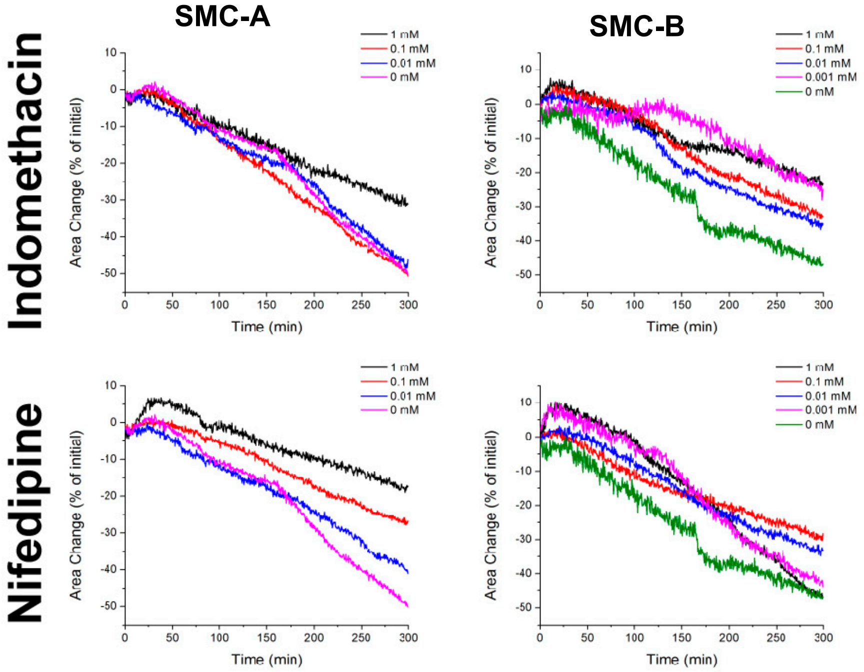

Figure S1. Time series of contraction by rings of commercially available smooth muscle cell (SMC)-A and SMC-B (PromoCell GmbH, Heidelberg, Germany), exposed to indomethacin and nifedipine. Exposure to the drug reduced contractions, particularly at higher concentrations, as expected. Moreover, each cell type had a different response to drugs, demonstrating our ability to detect differences between cell types.

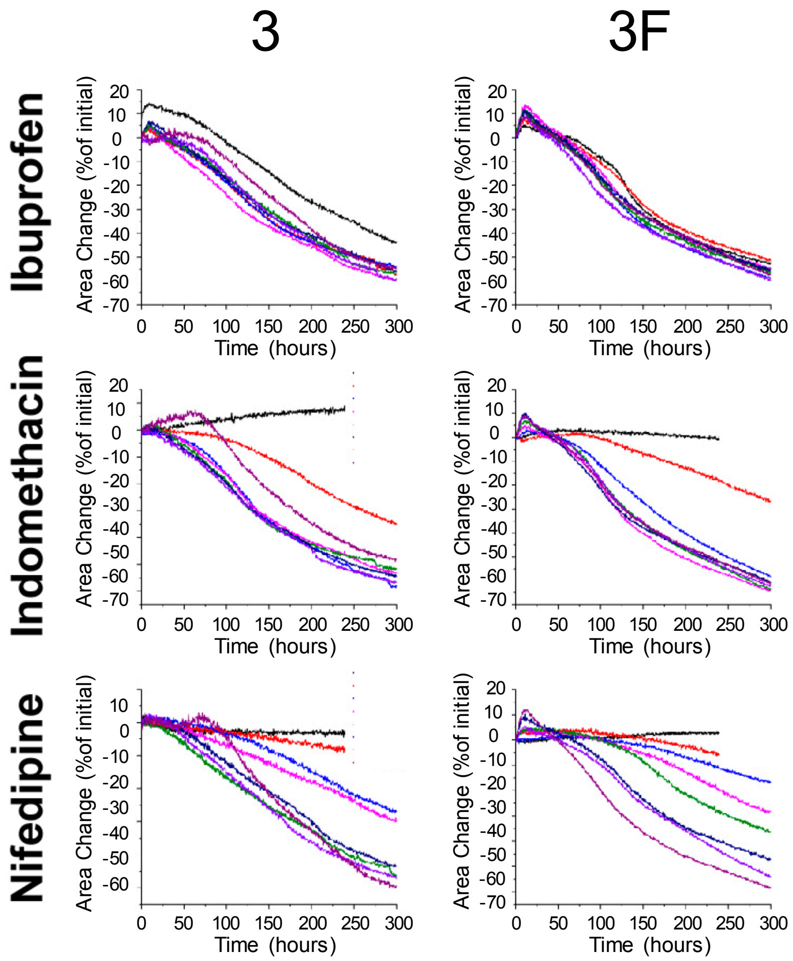

Figure S2. Comparison between the contraction of fresh and cryopreserved patient-derived myometrial smooth muscle rings over time. The contraction profiles of myometrial smooth muscle rings from Patient 3 exposed to varying concentrations of different compounds. The data show that the contractility responses are preserved after cryopreservation of the uterine tissue.
